# Prolonging Preservation or Assessment of Organ Quality—What is Key?

**DOI:** 10.3389/ti.2023.12174

**Published:** 2023-11-08

**Authors:** J. Eden, P. Dutkowski

**Affiliations:** ^1^ Section of HPB Surgery and Liver Transplantation, Department of Surgery, University of Groningen and University Medical Center Groningen, Groningen, Netherlands; ^2^ Department of Surgery and Transplantation, University Hospital Zurich, Zurich, Switzerland

**Keywords:** organ transplantation, vitrification, organ utilization, organ preservation, organ assessment

In their recent article [1], Han et al demonstrate successful vitrification and nanowarming of rat kidneys for up to 100 days, with subsequent transplantation in a rat transplant model. This is a milestone and represents unequivocally the longest out of body preservation time for a solid organ. The authors achieved such a remarkable result by a complex procedure, involving several important steps. First, loading of specific cryoprotective agents is performed by a short initial organ perfusion with ice blockers and iron oxide nanoparticles. Secondly, super rapid cooling is achieved with a cooling rate of 24°C per minute by a controlled freezer. Third, a deep temperature storage follows at −150°C, keeping the organ in a glassy state for up to 100 days. Fourth, super rapid and uniform rewarming is realized with a temperature increase of 78°C per min by a radiofrequency alternating electromagnetic field in a coil. Fifth, unloading of the cryoprotective agents is needed through another organ perfusion step. Finally, a final perfusion period is suggested with an assessment of organ quality under normothermic conditions (40 min, 37°C). The presented results suggest that such cryobanking is potentially more effective than earlier published work on super cooling at −6°C, which prolonged *ex situ* rat liver viability only up to 7 days [2], and human liver viability only up to 27 h [3].

As the authors state, the study is limited by the small size of the experimental groups in a rodent transplant model, and by a very short follow up of rat recipients, i.e., 1 month. It is therefore unclear if these experiments can be reproduced in large animal models or human organs, and whether a long-term high quality of transplanted cryopreserved organs can be assured. Undoubtedly though, successful cryobanking of human organs would completely change the field of organ transplantation in terms of scheduled procedures.

The disadvantage of this concept is on the other hand, that a static procedure, i.e., storage at −150°C, is unlikely to allow organ assessment. After cryobanking, the authors suggest therefore a short period of normothermic perfusion to check for organ quality. This is yet the most debatable point in the field, as reliable biomarkers are not available for kidneys and also not for livers, lungs, and hearts. Therefore, and in contrast to what the authors state, improved organ utilization will not necessarily increase by prolongation of preservation alone, but rather by improved assessment of organs before transplantation [4, 5].

There remains currently an inherent and unsolved difficulty in interpreting liver or kidney function during any kind of *ex situ* preservation, leading to the report of several so-called biomarkers, measured, for example, in circulating machine perfusates [6] or in produced bile and urine [7]. These include machine perfusate transaminanses, LDH, cytokines, danger proteins, lactate clearance, bile flow, bile pH, bile glucose, NGAL, creatinine, INR, factor V, or methacetin metabolism [6]. While most of these parameters are used during normothermic machine perfusion (NMP) of livers or kidneys, their potential to distinguish between good or bad organs remains very limited. This is based on the fact, that the above mentioned clinical markers are rather down-stream consequences of impaired organ function, but not cause related. In contrast, on the subcellular level, clear evidence points to mitochondria as the source of ischemia reperfusion injury in all solid organs [8]. Mitochondrial complex I and II injury, transition pore opening, release of mitochondrial DNA and danger signals, are therefore more upfront signals of organ cellular injury [8], and are also representative for an impaired organ function. Measurement of mitochondrial injury during *ex situ* machine perfusion has therefore gained attendance but needs further research [9, 10]. Besides, it is also unclear, which time period is needed for reliable organ assessment.

Another limitation is that the authors used healthy kidneys for the cryo-approach, i.e., kidneys without significant cold or warm ischemia. The realistic scenario in the transplant world is however the use of injured organs, which need additional transport to recipient centers in most cases. Successful cryobanking will require possibly already before vitrification organ pretreatment by machine perfusion, such as, for example, by initial hypothermic oxygenated perfusion, to minimize mitochondrial oxidative stress [11, 12], and to upload organ energy resources before cryobanking [13, 14].

Notably, the described procedure of cryobanking is the opposite to the alternative idea to keep organs in a functional status rather than to minimize metabolism. In fact, normothermic long-term kidney- or liver perfusions have been performed, but currently only for periods up to 2 days in kidneys [15] and for 7–10 days in livers [16, 17]. The shortcoming of normothermic perfusion systems is therefore an extensive effort and the need for sophisticated devices, due to *ex situ* simulation of the physiologic environment of human organs. The advantage of long-term normothermic perfusion is however the continued accessibility to the perfused organ with the option to treat and monitor outcome parameters, although it remains unsolved which parameters should be best tested (Figure 1).

**FIGURE 1:**
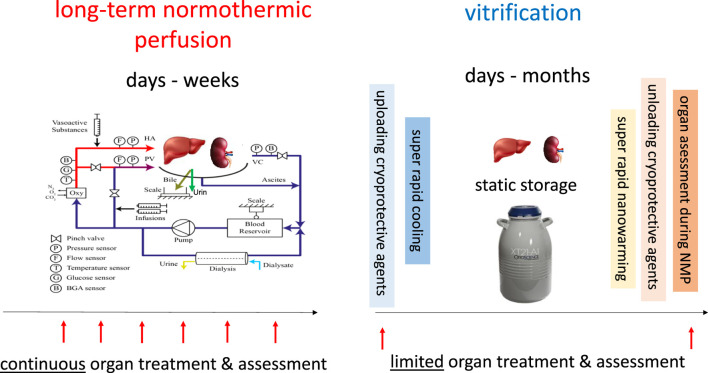
Comparison of long-term normothermic perfusion and cryobanking.

In conclusion it is unclear, whether long-term perfusion strategies or advanced cryobanking will have the highest impact on organ availability in the future, but a combination of both could be the best option. Both methods should therefore be further elaborated. True organ treatment and repair will likewise only be feasible at a functional state, i.e., with dynamic preservation procedures.

## Data Availability

The original contributions presented in the study are included in the article/supplementary material, further inquiries can be directed to the corresponding author.
